# Functional and Psychological Changes after Exercise Training in Post-COVID-19 Patients Discharged from the Hospital: A PRISMA-Compliant Systematic Review

**DOI:** 10.3390/ijerph19042290

**Published:** 2022-02-17

**Authors:** Amir Hossein Ahmadi Hekmatikar, João Batista Ferreira Júnior, Shahnaz Shahrbanian, Katsuhiko Suzuki

**Affiliations:** 1 Department of Sport Science, Faculty of Humanities, Tarbiat Modares University, Tehran 14117-13116, Iran; a.ahmadihekmatik@modares.ac.ir; 2 Federal Institute of Sudeste of Minas Gerais, Rio Pomba 36180-000, Brazil; jbfjunior@gmail.com; 3 Faculty of Sport Sciences, Waseda University, Tokorozawa 359-1192, Saitama, Japan

**Keywords:** COVID-19, coronavirus disease, SARS-CoV-2 virus, resistance and aerobic exercise, COVID-19 patients, rehabilitation, psychological changes, mental health

## Abstract

Millions of people worldwide are infected with COVID-19, and COVID-19 survivors have been found to suffer from functional disabilities and mental disorders such as depression and anxiety. This is a matter of concern because COVID-19 is still not over. Because reinfection is still possible in COVID-19 survivors, decreased physical function and increased stress and anxiety can lower immune function. However, the optimal exercise intensity and volume appear to remain unknown. Therefore, the current systematic review aimed to evaluate the effect of resistance or aerobic exercises in post-COVID-19 patients after hospital discharge. We conducted searches in the Scopus, SciELO, PubMed, Web of Science, Science Direct, and Google Scholar databases. Studies that met the following criteria were included: (i) English language, (ii) patients with COVID-19 involved with resistance or aerobic exercise programs after hospital discharge. Out of 381 studies reviewed, seven studies met the inclusion criteria. Evidence shows that exercise programs composed of resistance exercise (e.g., 1–2 sets of 8–10 repetitions at 30–80% of 1RM) along with aerobic exercise (e.g., 5 to 30 min at moderate intensity) may improve the functional capacity and quality of life (reduce stress and mental disorders) in post-COVID-19 patients. In addition, only one study reported reinfection of three subjects involved with the exercise program, suggesting that exercise programs may be feasible for the rehabilitation of the patients. A meta-analysis was not conducted because the included studies have methodological heterogeneities, and they did not examine a control group. Consequently, the results should be generalized with caution.

## 1. Introduction

The COVID-19 virus was recognized worldwide by the World Health Organization in 2020, and the virus was able to spread rapidly worldwide [1]. So far, more than 98 million people have been infected with COVID-19, resulting in nearly 2.2 million deaths [2]. The world is still facing this virus, and it continues to a serious threat to everyone.

Symptoms such as muscle pain, fatigue, and weakness are reported in post-COVID-19 patients [3]. The exact action mechanisms of COVID-19 on patients is not determined. However, muscle changes such as necrosis and muscle atrophy are also reported [4]. Overproduction of proinflammatory cytokines in hypermetabolic conditions is associated with the oxidative stress induced by the virus, which produces corrosive molecules that cause severe damage to myocytes [5]. Considering that metabolic and inflammatory disorders linked with sedentarism are common in the elderly [6], myopathy associated with COVID-19 may even be more dangerous in the elderly population. It is noted that older people with COVID-19 are more likely to experience significant damage in muscle structure, especially in the latter stages of the disease [7,8]. Sarcopenic muscles and adipose tissue produce myokines and adipokines, which stimulate inflammation and oxidative stress, leading to hyper catabolism [9]. Recent studies provided evidence of skeletal muscle dystrophic injuries in COVID-19 patients [7,10]. This muscle atrophy after hospital discharge may reduce the physical performance of the patients, affecting their health and quality of life [10]. This decrease in physical function can be worrying after discharge from the hospital. Recently, evidence suggests that people with COVID-19 will experience mental health problems after discharge from the hospital [11]. Additionally, the level of stress and anxiety after discharge from the hospital is high in these patients. So, Mental health changes, such as decreased sleep quality after hospital discharge, can cause a number of problems. This factor can negatively impact the functioning of the immune system and psychological changes [11].

As exercise training leads to morphological adaptations (e.g., increased number of contractile proteins and mitochondria) [12], it can be considered as a potential strategy to be performed to counteract the deleterious effects of COVID-19 on muscle tissue [13,14]. Exercise may also modulate the immune system [15,16,17,18]. It was recently revealed that the new COVID-19 strain (Omicron) was able to pose serious risks, and this marks the beginning of a new COVID-19 wave [19]. It was also shown that Omicron can bypass the innate immune system, so it can be said that there is a possibility of recurrence of COVID-19 survivors [19]. Thus, resuming an exercise training program after COVID-19 hospital discharge may optimize patients’ recovery. However, the optimal exercise intensity and volume appear to remain unknown. Evaluating this issue may help professionals who work with this population (e.g., physiotherapists) design better training programs for patients’ rehabilitation. Therefore, the current systematic review aimed to evaluate the effect of resistance or aerobic exercises in post-COVID-19 patients after hospital discharge.

## 2. Materials and Methods

The current study was carried out based on the guidelines and principles outlined by the PRISMA statement 2020 and checklist [20].

### 2.1. Search Strategies

The current systematic review was conducted from 26 November 2000 to 22 January 2021 based on a literature search of six electronic databases: Scopus, SciELO, PubMed, Web of Science, and Science Direct. Additionally, we searched Google Scholar to identify studies not found in the mentioned databases. The search terms were designed based on the following Medical Subject Heading (MeSH) keywords: “Resistance training”, “Resistance Exercise”, “Strength training”, “Weight training”, “Muscle strength”, “Aerobic training”, “Aerobic Exercise”, and “Muscle strength”; “Mobility” or “Physiological changes” or “Mental health”; “COVID-19 and mental changes” and “COVID-19 patient”

### 2.2. Inclusion and Exclusion Criteria

Legibility criteria were based on PICO using the following parameters: (i) Population—COVID-19 patients worldwide, (ii) Intervention—studies that evaluated patients with COVID-19 who performed resistance or aerobic training, (iii) Comparison—pre-and post-training measurements, and (iv) Outcome—objective measurement of muscle strength (i.e., isokinetic strength, isometric strength and maximal strength [one-repetition maximum, 1RM], and sit-to-stand test), muscle hypertrophy (ultrasound, magnetic resonance imaging or computed tomography), functional capacity (maximal functional capacity, 6 min walk test, functional independence measure, short physical performance battery, maximal heart rate, and gait speed). Additionally, only studies published in English were selected.

### 2.3. Data Extraction

The following data were extracted: authors name; year of publication; sample size; participants characteristics (age, sex, and training status); training characteristics; assessed parameters (e.g., muscle size and strength, functional capacity, quality of life, and fatigue); and main outcomes (e.g., maximum muscle strength, muscle size, functional capacity, quality of life, and fatigue). Studies’ limitations were also examined.

### 2.4. Study Quality and Risk of Bias Assessment

Two authors (AAH and SSH) independently assessed the risk of bias using the risk of bias tool for non-randomized studies (ROBINS-I) [21]. Then, traffic light and weighted summary risk-of-bias plots for non-randomized included studies were produced by the risk-of-bias visualization (robvis) online tool [22]. Finally, the agreement between the two authors (AAH and SSH) on study quality and risk of bias assessment was 70 to 80%, respectively. Any discrepancies were resolved through discussion.

## 3. Results

### 3.1. Included Studies

**Figure 1:**
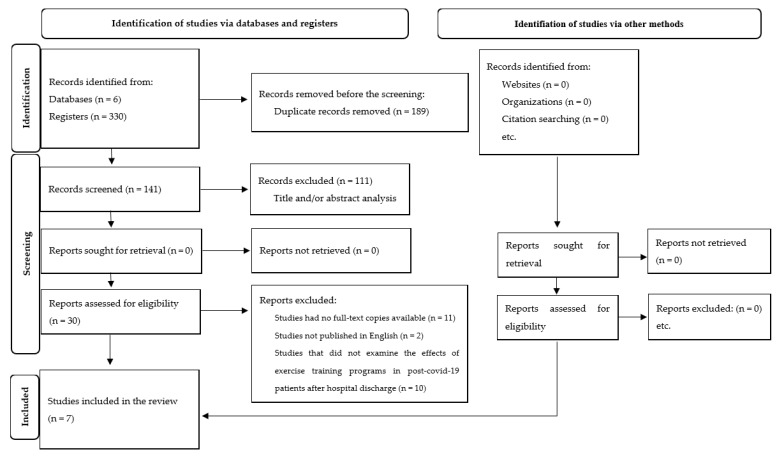
Diagram flow of outcomes of the review.

The search strategy retrieved 330 records, and 189 studies were omitted because they were duplicates. Then, out of the remaining 141 studies, 111 studies were excluded after screening and title and/or abstract analysis. Then, out of the remaining 30 studies, 23 studies were excluded for the following reasons: (i) 11 studies had no full-text copies available, (ii) 2 studies were not published in English, (iii) 10 studies did not examine the effects of resistance or aerobic training programs in post-COVID-19 patients after hospital discharge. At the end of the process, seven publications meeting the eligibility criteria were included for analysis. Figure 1 depict the diagram flow of outcomes of the review.

The characteristics of the participants are summarized in Table 1. A total of 286 (188 men and 98 women) participants were examined. The number of participants in these studies ranged from 7 to 115. One study evaluated only men, and the other six studies were a combination of men and women. The age of the participants in the present study ranged from 20 to 84 years. All subjects were diagnosed with COVID-19 and started the exercise training intervention after hospital discharge. The studies did not inform the physical fitness level or the exercise experience of the subjects.

The characteristics of aerobic and resistance training interventions are reported in Table 2. The intervention programs ranged from 10 sessions to 12 weeks. Several types of aerobic training were employed (e.g., cycle ergometer, steps, walking, and treadmill running). Aerobic exercise duration ranged from 5 to 30 min; however, three studies did not report the exercise duration. Three studies reported the aerobic exercise intensity based on maximum heart rate (at 40–60%) or based on peak work rate (at 50%). The other two studies designed the intensity of aerobic exercise based on the rate of perceived exertion, which ranged from 4 to 6 of 10. Additionally, aerobic exercise intensity was not reported by two studies. Concerning resistance training, exercise intensity varied from 30 to 80% of 1-RM. Only one study did not inform the resistance exercise intensity. The average exercise intensity was between 50 and 70% of 1-RM. The number of repetitions ranged from 8 to 20 repetitions, and the mean repetitions were between 8 and 12 repetitions. In addition, the number of sets in resistance training varied from two to three sets. Two studies did not report the number of sets and repetitions performed by the subjects. Furthermore, the studies did not inform the rest time between sets and resistance exercises. The resistance training protocol included upper body and lower body exercises; however, three studies did not report the exercises’ number performed or the muscle group trained.

Regarding limitations, the included studies did not report the severity of the previous disease, the physical fitness level, and the exercise experience of the patients. Other significant diseases of the patients involved in the analysis were also not informed.

### 3.2. Outcomes

The parameters examined by the studies are reported in Table 2. Only one study examined muscle hypertrophy. Four studies assessed strength-related changes through the handgrip strength test or the sit-to-stand testing. An objective measure of functional capacity was reported by five studies (gait speed test or 6 min walk test). Two studies assessed performance through functional tests (e.g., Barthel index and short physical performance battery). One and three studies assessed fatigue and quality of life, respectively. All studies showed that the mentioned parameters improved after exercise training interventions (Table 2). These studies found that after rehabilitation training, the quality of life improves and the level of anxiety decreases, which can eventually be said to cause positive psychological changes.

### 3.3. Quality and Risk of Bias Assessment

**Figure 2:**
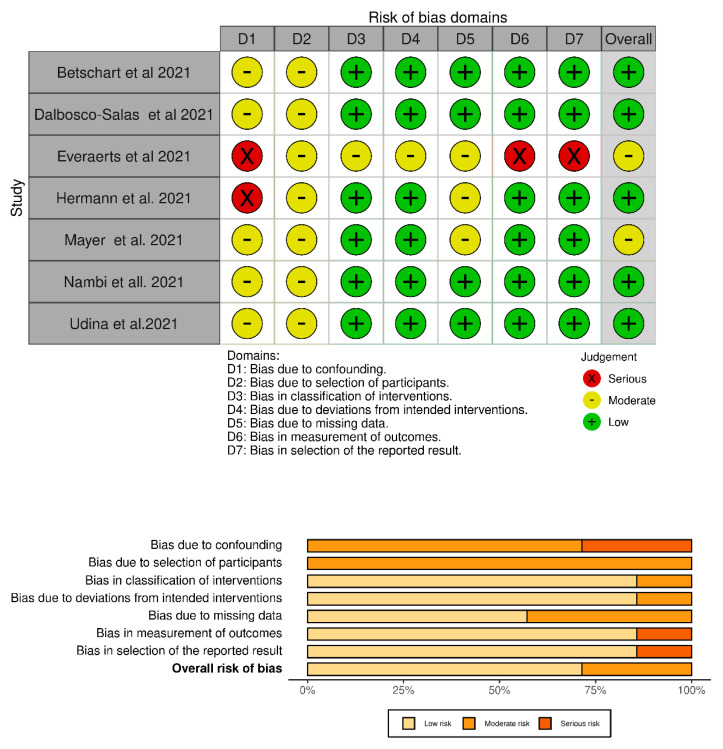
Risk of bias assessment of the randomized trials.

Figure 2 summarize the results of the risk of bias assessment for the non-randomized controlled trials evaluated in the present systematic review. The risk of bias was low in five studies [23,24,25,26,27], and there were some concerns in two studies [28,29].

## 4. Discussion

The present systematic review aimed to analyze the scientific evidence on functional and psychological changes after exercise training in post-COVID-19 patients discharged from the hospital. It was assumed that regular exercise may play a significant role in the health status (psychological and physiological) of patients after hospitalization [30]. The analyzed studies have shown that performing resistance and aerobic exercise after hospital discharge may improve functional and mental capacity.

The guidelines for exercise prescription aiming at health promotion and rehabilitation recommend performing both resistance and aerobic exercise [31,32]. Resistance exercise is of great importance among the approaches composing a training program. From a clinical perspective, the health benefits of resistance exercises are well-proven by over 30 years of research [33]. In summary, meta-analyses of short-term clinical exercise studies show that resistance training increases skeletal muscle mass and strength and improves the ability to perform daily life activities [34,35]. Resistance training is also shown to reduce the symptoms of depression and anxiety [36]. It was reported that resistance training alone or combined with aerobic exercise might increase muscle performance and improve quality of life [13,35,37,38,39]. Compared to aerobic exercise alone, resistance training combined with aerobic exercise may even induce higher effects on emerging health conditions, such as the prevention and/or treatment of sarcopenia and physical function maintenance [40,41]. Our recent epidemiological studies showed that a combination of both exercises might be useful in preventing and/or managing several common chronic diseases [23,24].

The results of the present systematic review confirm these reports. Nambi et al. [24] examined resistance training combined with low- or high-intensity aerobic exercise in post-COVID-19 patients. The authors found a higher increase in handgrip strength, muscle growth, and quality of life for the low-intensity aerobic exercise group when compared to the high-intensity aerobic exercise group. Exercise intensity and volume were considered the main parameters for exercise prescription. Therefore, improving the quality of life along with physiological changes can be effective in returning to normal living conditions. Numerous studies showed that high-intensity aerobic and resistance training or long exercise sessions (≥1.5 h) may lead to temporary immune system suppression [14,42,43,44]. Due to the nature of COVID-19 disease, in which the immune system is involved, it is recommended to avoid immunosuppression induced by exercise. A recent study reported that three patients refused to continue the exercise protocol due to recurrence of infection [23]. The aerobic exercise was performed at low- and moderate-intensity in the continuous and interval mode, respectively (Table 1). According to Everaerts et al. [29], the training program was interrupted in four patients due to interfering medical problems (myasthenia gravis, lumbar discus hernia, severe cognitive dysfunction). Another study showed that a short training period (i.e., 10 days) induced significant improvements in physical performance in post-COVID-19 patients [27]. Exercise intensity ranged from 30 to 80% of 1RM for resistance exercises and from 3 to 5 of modified Borg scale for the aerobic exercise. According to the authors, there was no reinfection during the training period. Hermann et al. [25] also reported that none of the patients died or had to be taken back to the hospital after performing resistance and aerobic exercises. The aerobic exercise was performed at moderate intensity, 20 repetitions with the maximum tolerated load for the resistance exercises were completed. Furthermore, training sessions ranged from 30 to 90 min in all analyzed studies. Taken together, these studies suggest that resistance and aerobic exercises are feasible approaches to optimize recovery from COVID-19.

The current systematic review also showed that training programs composed of resistance and aerobic exercises increased muscle strength, reduced activity-induced shortness of breath and fatigue index, and improved functional independence and quality of life in patients after hospital discharge by COVID-19 [45]. Moreover, the remote supervision of exercise training seems to be an effective strategy for rehabilitating patients after COVID-19 infection [24]. However, the current study is not without limitations. It is noted that none of the analyzed studies examined a control group. The included studies also did not provide the severity of the physical fitness level and the exercise experience of the patients. Other significant diseases of the patients involved in the analysis were also not informed. Additionally, it was not possible to perform a meta-analysis because the included studies have methodological heterogeneities. Mayer et al. [24]. also found that resistance and aerobic exercise can cause both positive physiological and psychological changes. Consequently, these results should be generalized with caution. Therefore, future high-quality randomized controlled trials evaluating the effects of exercise programs after hospitalization by COVID-19 are needed.

In conclusion, the present systematic review showed that exercise programs composed of resistance exercise (e.g., 1–2 sets of 8–10 repetitions at 30–80% of 1RM) along with aerobic exercise (e.g., 5 to 30 min at moderate intensity) seem to be feasible for the rehabilitation of post-COVID-19 patients.

## Figures and Tables

**Table 1:** Training interventions characteristics of the included studies (n = 6).

Eligible Study	Subjects	Training Protocol	Training Period	Days/Week
Betschart et al., 2021	N = 12 (4 females and 8 males)	30 min of aerobic cycle exercise (two sessions of continuous mode [20–30% peak WR] followed by two sessions of interval mode [warm-up 4 min at 15% peak WR 4 × 4 min at 50% peak WR and 3 × 3 min at 20–30% peak WR, and cooling-down 3 min at 15% peak WR]) combined with six RE (three sets of 10–12 repetitions at 50–85% of 1RM).	8–12 weeks	2x
Dalbosco-Salas et al., 2021	N = 115 (44 males and 66 females; 57 post-hopitalization and 58 non-hospitalized).	Home-based exercise training is composed of warm-up (5 min), breathing exercises (3 min), aerobic and/or strength exercises (20–30 min), and stretching (5 min). Volume and intensity of aerobic and RE were not reported.	9 weeks	2–3x
Everaerts et al., 2021	N = 22 adults (7 females and 15 males) with muscle strenght or 6 min walk test below 70% of the predicted values	Aerobic exercise (treadmill, cycle ergometer, arm ergometer, and stair climbing or step), next to RE (leg press and chest press). The program started at 60–75% of maximal individual performance. Interval training was implemented if the patient was not able to cycle ≥10 min on 80% VO_2peak_. Exercise intensity and duration increased progressively based on symptom scores (target Borg dyspnoea and fatigue score 4–6/10). The volume of the aerobic and resistance exercise was not reported.	12 weeks	3x
Hermann et al., 2021	N = 28 (15 female and 13 males; 112 in the post-ventilation group and 16 in the non-ventilation froup).	Aerobic exercise (outdoor walking, or cycle ergometer, and criteria for stopping or reducing exercise intensity was SpO2 <88%, Borg scale >6 or/and reaching their submaximal heart rate, duration was not informed) followed by RE (3 sets of 20 repetitions with the maximum tolerated load, number of exercises was not informed).	3–4 weeks	5–6x
Mayer et al., 2021	N = 32 males (14 female and 18 males; 22 in the in-person treatment group and 10 in the telehealth treatment group).	In-person program: Aerobic exercise (15–30 min at an intensity of 4–6 on the modified Borg scale), RE (three sets of 10–15 repetitions at RPE of 5–6 of 10), breathing and mindfulness techniques. Telehealth program: walking (30 min at an RPE ≤ 4), strengthening exercises (not detailed), and breathing techniques	8 weeks	3–4x
Nambi et al., 2021	N = 76 males with post-COVID-19 sarcopenia (38 in the low-intensity aerobic group and 38 in the high-intensity aerobic group)	11 RE (3 sets of 10RM, 60 s of rest interval, combined with 30 min of low (40–60% of HRmax) or high-intensity (60–80% of HRmax) aerobic exercise (20 min on treadmill and 10 min on a cycle ergometer).	8 weeks	4x
Udina et al., 2021	N = 33 (19 females and 14 males; 20 in the post-ICU group and 13 in the non-ICU group)	2–4 RE (1–2 sets of 8–10 repetitions at 30–80% of 1RM) and 5–15 min of endurance exercise (cycle ergometer, steps or walking at an intensity of 3–5 of modified Borg scale) and two balance exercises (walking with obstacles, changing directions or on unstable surfaces).	10 days	7x

RE: resistance training. RM: repetition maximum. HRmax: maximal heart rate. WR: work rate. ICU: intensive care unit. VO2peak: peak oxygen uptake.

**Table 2:** Outcomes of the reviewed studies (n = 6).

Studies	Parameters	Results
Betschart et al., 2021	6 min walk test, health-related quality of life	6 min walk test (m): mean change of 88 Health-related quality of life: improved from a mean of 65% to 81%
Dalbosco-Salas et al., 2021	1 min sit-to-stand test, health-related quality of life, fatigue	1 min sit-to-stand test (number of repetitions): improved from a mean of 16.8 to 26.5 in the post-hospitalization group and from a mean of 24.2 to 32.2 in the non-hospitalization group Health-related quality of life: improved from a mean of 37.1 to 54.1 in the post-hospitalization group and from a mean of 41.9 to 63.3 in the non-hospitalization group Fatigue (VAS): improved from a mean of 3 to 1 in the post-hospitalization group, and it was not altered in the non-hospitalization group (mean of 1.5 to 1).
Everaerts et al., 2021	6 min walk test, handgrip strength, quadriceps force, cardiopulmonary exercise test, HADS and MoCA	6 min walk test (m): improved from a mean of 453 to 549 after 6 weeks and to 605 after 12 weeks Handgrip strength improved to 104% of the predicted values Quadriceps force improved to 74% of the predicted values VO_2peak_ (ml.kg^−1^.min^−1^) improved from a mean of 16 to 20 Anxiety score: a non-significant change from 5 to 6 Depression score: 3 for both baseline and post-training period MoCA: a non-significant change from 25 to 28
Hermann et al., 2021	6 min walk test	6 min walk test (m): mean change of 145.4 ± 59.1 in the post-ventilation group and 118.5 ± 89.8 in the non-ventilation group
Mayer et al., 2021	SPPB global score, 6 min walk test, handgrip strength, chair stand test, health-related quality of life, gait speed	SPPB global score: improved from a mean of 7.8 to 10.1 6 min walk test (m): mean change of 101 ± 93 Right handgrip strength: improved from a mean of 25.9 to 29.8 Chair stand test (s): improved by a mean of 4.9 ± 6.1 Health-related quality of life (VAS): improved from a mean of 72 to 83.4 There was no difference between the in-person and telehealth groups Gait speed (m/s): mean average 0.22 m per second, and TUG improved by 3.2 s, respectively
Nambi et al., 2021	Handgrip strength; cross sectional area of arm, thigh and calf; and quality of life	Handgrip strength: ↑ 10.9% in the low-intensity group and 4.5% in the high-intensity group Arm cross-sectional area: ↑ 5.3% in the low-intensity group and 4.8% in the high-intensity group Thigh cross-sectional area: ↑ 7.8% in both low- and high-intensity groups Calf cross-sectional area: ↑ 10.1% in the low-intensity group and 10.3% in the high-intensity group Quality of life: ↑ 20.4% in the low-intensity group and 4.8% in the high-intensity group
Udina et al., 2021	SPPB global score, gait speed, chair-stand time, and Barthel Index.	SPPB global score: mean change of 4.4 ± 2.1 in the post-ICU group and 2.5 ± 1.7 in the non-ICU group Gait speed (m/s): mean change of 0.4 ± 0.2 in the post-ICU group and 0.2 ± 0.1 in the non-ICU group Chair-stand time (s): mean change of −15.3 ± 16.9 in the post-ICU group and −12.2 ± 17.6 in the non-ICU group Barthel index: mean change of 18.2 ± 12.4 in the post-ICU group and 18.8 ± 14.01 in the non-ICU group

HADS: Hospital Anxiety and Depression Scale. MoCA: Montreal Cognitive Assessment. SPPB: Shortort Physical Performance Battery. STST: sit-to-stand test. VAS: visual analogue scale. VO_2peak_: peak oxygen uptake.

## Data Availability

Not applicable.
